# Purification and Characterization of Immunomodulatory Peptides from Hydrolysates of *Thunnus albacares* Dark Muscle

**DOI:** 10.3390/foods14061068

**Published:** 2025-03-20

**Authors:** Cunkuan Shen, Yuanfang Xu, Jinxin Yan, Xiangyang Qi, Shaoqian Cao, Hua Yang

**Affiliations:** College of Biological and Environmental Science, Zhejiang Wanli University, Ningbo 315100, China; shenck2022@zwu.edu.cn (C.S.); xuyuanfang0725@163.com (Y.X.); yanjinxin@zwu.edu.cn (J.Y.); qixiangyang@zwu.edu.cn (X.Q.)

**Keywords:** *Thunnus albacares*, peptides, immunomodulatory activity, molecular docking

## Abstract

Food-derived bioactive peptides have attracted considerable research interest and are increasingly utilized as functional ingredients in the food industry. In this study, the immunomodulatory peptides were isolated and purified from *Thunnus albacares* (*T. albacares*) enzymatic hydrolysates of muscles using gel chromatography and RP-HPLC, and their amino acid sequences were identified via LC-MS/MS. A total of six peptides were selected based on their affinity to toll-like receptors. Subsequently, these peptides were synthesized to confirm the immunomodulatory activities in vitro. Among all the tested peptides, two peptides, HDCDLLR and YGSVELDELGK, significantly enhanced cell proliferation and phagocytosis and increased the production of tumor necrosis factor-α (TNF-α), nitric oxide (NO), and interleukin-6 (IL-6). Molecular docking analysis indicated that these two peptides could stably bind to the receptors through hydrogen bonds and electrostatic and hydrophobic interactions. These findings suggested that peptides from enzymatic hydrolysates of *T. albacares* could be promising candidates for developing immunomodulatory agents in functional foods.

## 1. Introduction

The immune system plays a crucial role in preventing various infectious diseases by identifying and killing pathogens, aging cells, and tumor cells. It comprises two subsystems: the innate immune system and the adaptive immune system. Innate immunity offers the first line of defense, delivering non-specific protection via mucosal tissue, skin, bone marrow, or inflammatory components, with macrophages playing a pivotal role in phagocytosis. Meanwhile, adaptive immunity forms the body’s second line of defense, with T and B lymphocytes orchestrating target responses. Many factors, such as unhealthy lifestyle, stress, pathogens, and foreign antigens, can significantly impact its function [1]. The cooperative actions of humoral and cell-mediated immune responses effectively counter these threats, highlighting immunostimulatory agents as a potential strategy to manage immunity-related diseases.

Bioactive peptides are specific protein fragments that exert a beneficial physiological effect on the human body, influencing health beyond basic nutrition [2]. They generally consist of sequences of 3–20 amino acids, exhibiting diverse biological activities that are primarily determined by their amino acid sequences and the source protein [3,4]. These peptides can be extracted from food proteins—both animal [5] and plant sources [6]—via enzymatic hydrolysis, microbial fermentation, and thermal processing methodologies [7]. They offer a spectrum of health benefits, such as antimicrobial, antithrombotic, antioxidative, and antihypertensive effects, alongside their capacity to modulate the immune system [8].

Naturally occurring bioactive peptides like immune active peptides are believed to modulate immune functions and thus continue to attract the interest of researchers in developing nutraceuticals, functionals foods, and therapeutic agents [9]. These peptides enhance immune surveillance by promoting antibody synthesis, regulating T and B lymphocyte populations, and increasing the phagocytic activity of natural killer (NK) cells and macrophages. It has been reported that these immunomodulatory peptides may interact with opioid receptors on the surface of immune cells, thereby influencing the peripheral immune system through central opioid regulation [10]. Additionally, the efficacy of these peptides are mostly controlled by their structural conformation, as influenced by their amino acid sequence and length [11,12]. Exploring the interaction between peptides and receptors on macrophages, as well as understanding their structural characteristics, is crucial for elucidating their immunomodulatory mechanisms.

Yellowfin tuna (*Thunnus albacares*) is a highly nutritious natural resource containing high-quality proteins, balanced fatty acids, and diverse bioactive compounds [13]. Over the past decades, the growing consumption demand for this species in human diets has led to massive by-product generation, such as dark muscle. Despite their high protein content, these by-products are frequently discarded, leading to considerable waste and environmental pollution, particularly in developing nations [14]. While these residues have traditionally been converted into fish meal for animal feed [15], there is a pressing need for innovative processing methods to transform these fish by-products into more economically viable and marketable products [16]. In recent years, these by-products have also caught special attention from researchers for being a cost-effective and sustainable source of bioactive compounds, such as bioactive peptides [17]. A number of studies on the isolation, purification, and characterization of bioactive peptides of *T. albacares* have been reported [18,19,20]. However, a comprehensive characterization of *T. albacares* by-products, especially immunomodulatory peptides, has not been reported so far.

The objective of this study was to isolate, purify, and identify immunomodulatory peptides from *T. albacares* dark muscle hydrolysates. Initially, the amino acid sequences of potential immunomodulatory peptides were determined through chromatography and mass spectrometry methods. These peptides were then screened using molecular docking approaches, synthesized to validate their immunomodulatory effects, and further examined to elucidate their interaction mechanisms with toll-like receptors 2 (TLR2) and TLR4/MD-2 receptors. This study would contribute to the high-value utilization of *T. albacares* resources and provide a theoretical foundation and technical support for its application in the food industry.

## 2. Materials and Methods

### 2.1. Materials

Yellowfin tuna (*Thunnus albacares*) muscle tissues were purchased from Ningbo Today Food Co., Ltd. (Zhejiang, China) and stored at −60 °C until use. Trypsin, Sephadex G-25, Lipopolysaccharide (LPS), Neutral red uptake, IL-6, and TNF-α ELISA kits were purchased from Solarbio Co., Ltd. (Beijing, China). The total Nitric Oxide Assay Kit was purchased from Beyotime Biotechnology Co., Ltd. (Shanghai, China), and the Cell Counting Kit-8 (CCK-8) was purchased from APExBio (APExBio Technology LLC., Houston, TX, USA). Fetal bovine serum (FBS) was purchased from Gibco Co. (Gibco BRL, Grand Island, NY, USA). Dulbecco’s Modified Eagle’s medium (DMEM) was purchased from Shanghai iCell Bioscience Co., Ltd. (Shanghai, China). Acetonitrile (ACN), formic acid (FA), and trifluoroacetic acid (TFA) were of HPLC-grade (Fisher chemical). All other chemical reagents used were of analytical grade.

### 2.2. Preparation of Thunnus Albacares Protein Hydrolysate (TAPH)

The TAPHs were prepared according to the method of Xu et al. [21]. Briefly, a muscle sample of 100 g was thoroughly homogenized using a HZ-300 laboratory homogenizer (JOANLAB, Huzhou, China) for 3 min at 10,000 rpm with 5 volumes of water in the flask. The pH of the mixture was adjusted to 8.0 using 1M NaOH. Then, trypsin was added to give an enzyme/substrate ratio of 6000 U/g protein and incubated for 5 h at 37 °C with magnetic stirring. After finishing the hydrolysis reaction, the mixture was boiled for 15 min to inactivate the protease, then cooled to room temperature, and centrifuged at 11,000× *g* for 20 min. The resulting supernatant was collected, lyophilized using a freeze-drier (SCIENTZ-10N, Xinzhi, Ningbo, China), and stored at −60 °C for further purification and immunological activity testing.

### 2.3. Peptide Fractionation by Gel Filtration Chromatography

The lyophilized TAPHs were fractionated using gel filtration chromatography based on the method described by He et al. [22] with minor modification. Firstly, 200 mg of TAPHs was dissolved in 2 mL of ultrapure water and filtered using a water-soluble Millipore filter (0.45 μm, Merck Millipore, Dublin, Ireland). Afterwards, 1 mL of this solution was loaded onto a pre-packed Sephadex G-25 column (2 × 50 cm) that was pre-equilibrated with ultrapure water. The flow rate was set to 1.5 mL/min, and the eluent was collected every 5 min while measuring the absorbance at 220 nm. Each fraction was pooled, lyophilized and stored for subsequent determination of their immunological activity.

### 2.4. Reversed-Phase High Performance Liquid Chromatography (RP-HPLC)

The fraction exhibiting the highest proliferative activity (methodology detailed in Section 2.8.2) from gel filtration was further purified using RP-HPLC. Briefly, the freeze-dried fraction was weighed and dissolved in ultrapure water to obtain a final concentration of 5 mg/mL, then filtered through a 0.22 μm-pore-size filter membrane. Subsequently, 500 μL of this solution was purified on a RP-HPLC system (Agilent 1100, Palo Alto, CA, USA) equipped with an Agilent Zorbax SB-C18 semi-preparative column (250 × 9.4 mm 10 μm). The flow rate was set to 1 mL/min, with 0.1% TFA in water and 0.1% TFA in ACN as mobile phase A and B, respectively. The elution was performed with a multi-step linear gradient as follows: 1–5% B in 2 min, 5–25% B in 30 min, 25–40% B in 5 min, and 60–95% A in 25 min. Absorbance of the eluent was monitored at 214 nm. Fractions were collected according to the elution peaks and then concentrated using a Savant SPD121P-230 SpeedVac (Thermo Electron Corporation, Milford, MA, USA). These concentrated fractions were further lyophilized and stored for subsequent determination of their immunological activity.

### 2.5. Peptide Sequence Identification by Using Liquid Chromatograph/Tandem Mass Spectrometry (LC–MS/MS)

Peptide sequences from the HPLC fractions were identified using an EASY-nLC 1200 nano-liquid chromatography handling system (Thermo Fisher Scientific, Waltham, MA, USA) coupled with a Q Exactive™ Hybrid Quadrupole-Orbitrap™ Mass Spectrometer (Thermo Fisher Scientific, USA) with an ESI nanospray source. Before the analysis, the peptide solution was first desalted using a C18 cartridge (Empore, 3M, St Paul, MO, USA) and then loaded onto a C18 analytical column (1.9 μm, 150 μm × 15 cm, 100 Å, Dr. Maisch GmbH, Ammerbuch-Entringen, Germany). The procedure was performed at a flow rate of 600 nL/min, and the gradient was set as follows: 4% solvent B in solvent A increasing linearly to 95% B over 60 min, where solvent A was 0.1% FA in water, and solvent B was 80% ACN, 20% water with 0.1% FA. The mass spectrometry data was acquired in a data-dependent manner in positive ion mode. MS survey scans were recorded from *m*/*z* 100 to 1500 at a resolution of 70,000 and an automatic gain control (AGC) of 3 × 10^6^. The top 20 precursors were selected for MS/MS acquisition at a 17,500 resolution and 1 × 10^5^ AGC. The raw MS data were analyzed and searched using PEAKS Studio10.6 software (Waterloo, ON, Canada). The parameters were set as follows: the protein modifications were carbamidomethylation (C) (fixed), oxidation (M) (variable), and Acetylation (N-term); the enzyme specificity was set to non-specific; the maximum missed cleavages were set to 2; the precursor ion mass tolerance was set to 20 ppm; and MS/MS tolerance was 0.02 Da. Only those peptides with average local confidence (ALC) scores of 90 or higher were chosen for further analysis.

### 2.6. Molecular Docking of Peptides with TLR2 and TLR4/MD2

The 3D structure of the selected peptides was generated using Chem3D software (v17.0) with minimal energy. Crystal structures of TLR2 (PBD code: 1FYW) and TLR4/MD2 (PBD code:5IJD) were obtained from RSCB PDB Protein Data Bank (https://www.rcsb.org, accessed on 18 August 2024). The molecule docking was performed using the flexible docking tool of AutoDock 4.2.6 software. A total of 100 docking runs of each molecule were performed, where the docking conformation with the lowest interface energy was chosen according to the scores and binding-energy value.

### 2.7. Peptide Synthesis

The potential immunologically active peptides identified through molecular docking analysis were chemically synthesized using the solid-phase procedure by Hefei Bank-peptide Biotechnology Co., Ltd. (Hefei, China). The molecular masses and purity (>98%) of these synthetic peptides were determined using RP-HPLC coupled with LC-MS. Peptides were dissolved in phosphate-buffered saline (PBS, 1.47 mM KH_2_PO_4_, 8.10 mM Na_2_HPO_4_, 2.68 mM KCl, 137 mM NaCl, pH 7.4) and stored at –80 °C until analysis.

### 2.8. RAW 264.7 Cellular Immunomodulatory Activity Assays

#### 2.8.1. Cell Culture

Murine macrophage cells (RAW 264.7) were purchased from the Chinese Academy of Medical Sciences, Shanghai institute Cell Bank (Shanghai, China). The cells were cultured in full DMEM supplemented with 10% FBS and maintained at 37 °C and 95% humidity in a 5% CO_2_ incubator (WCI-40, Wiggens, Beijing, China). Cells were passaged every two days at approximately 80% confluence and used for subsequent cellular activity experiments at passage 5–15.

#### 2.8.2. Cell Proliferation Assay

The effects of hydrolysate and peptides on cell proliferation were measured using the CCK-8 kit, following the manufacturer’s instructions. This assay is based on the bio-reduction of a water-soluble tetrazolium salt to a colored formazan product by metabolically active cells, thus reflecting cell viability rather than the direct counting of proliferation [23]. RAW 264.7 cells were first seeded into 96-well plates (approximately 1 × 10^5^ cells/mL) and incubated for 24 h. Then, the cells were treated with hydrolysates or peptides at a concentration of 200 μg/mL (prepared by dissolving lyophilized powder in PBS) and allowed to proliferate for another 24 h. LPS (2 μg/mL, dissolved in DMEM supplemented with 10% FBS) and DMEM supplemented with 10% FBS alone were used as the positive and negative control, respectively. CCK-8 solution (10 μL) was added to the wells, incubated for another 4 h, and measured at OD 450 nm using a microplate reader (SpectraMax, Molecular Devices, San Jose, CA, USA). LPS-treated cells were counted as 100%. The cell proliferation rate was calculated using the following equation:

$$Proliferation\;rate\;(\%)\;=(A_{1}-A_{0})/A_{0}\times 100$$

where *A*_1_ is the OD value of hydrolysates or peptides and *A*_0_ is the OD value of negative control.

#### 2.8.3. Phagocytosis Assay

The effects of hydrolysate and peptides on the phagocytic ability of RAW 264.7 cells were measured according to the method described by Repetto et al. [24] with slight modification. Cells were seeded into 96-well plates (approximately 1 × 10^5^ cells/mL) and incubated for 24 h. The supernatant was removed, and cells were treated with the tested samples and LPS (2 μg/mL). After another 24 h incubation, 100 μL of 0.09% neutral red solution (prepared in PBS) was added and incubated for an additional 2 h. Cells cultured in DMEM were used as the control. Then, the cells were washed with 150 μL of PBS, and 100 μL of cell lysis buffer (50% ethanol and 50% acetic acid) was added. The absorbance was recorded at 520 nm. The cell phagocytosis rate was calculated using the following equation:

$$Phagocytosis\;rate\;(\%)\;={(A}_{1}-A_{0})/A_{0}\times 100$$

where *A*_1_ is the OD value of hydrolysates or peptides and *A*_0_ is the OD value of the negative control.

#### 2.8.4. Griess Assay

The effects of hydrolysates and peptides on the nitric oxide (NO) production of macrophages were measured using a colorimetric assay based on the Griess reaction [25]. Briefly, RAW 264.7 cells (1 × 10^4^ cells per well) were seeded in 96-well plates and incubated in the absence or presence of samples at 37 °C for 24 h, while LPS (2 μg/mL) was used as a positive control. The culture supernatant reacted with the Griess reagent at room temperature for 10 min, and then the nitrite concentration was determined by measuring the absorbance at 550 nm. NO concentration was calculated using a series of standard solutions of sodium nitrite dissolved in DMEM medium supplemented with 10% FBS. The standard curve was prepared using concentrations of 0, 1, 2, 5, 10, 20, 40, 60, and 100 μM.

#### 2.8.5. Cytokine Assays

RAW 274.7 cells were seeded into 6-well plates (approximately 1 × 10^5^ cells/mL) and incubated for 24 h, followed by treatment with the selective peptide solutions dissolved in DMEM medium for another 24 h. The supernatant was removed and the concentration of IL-6 and TNF-α were determined using the ELISA kit according to the manufacturer’s instructions.

### 2.9. Statistical Analysis

All the experiments were performed in triplicates, and data were expressed as means ± standard deviations. Data were analyzed using SPSS, version 22 (IBM, Armonk, NY, USA) and visualized with GraphPad Prism, version 8.0 (GraphPad, San Diego, CA, USA). A *p*-value less than 0.05 was considered statistically significant.

## 3. Results and Discussion

### 3.1. Preparation of TAPHs

In the present study, *Thunnus albacares* protein hydrolysates (TAPHs) were used as the source of immunomodulatory peptides. Due to the specific cleavage patterns of different proteases, a vast array of peptides with different lengths and amino acid sequences could be generated. Previous work in our laboratory investigated the effects of five proteases (papain, trypsin, alcalase, neutrase, and pepsin) on the immunomodulatory activity of TAPHs. The findings revealed that TAPHs prepared with trypsin exhibited the most potent immunomodulatory effects on RAW 264.7 cells [17]. Consequently, this hydrolysate was selected for subsequent peptides separation and isolation in this study.

### 3.2. Purification of Immunomodulatory Peptides from Trypsin Hydrolysates of T. albacares Dark Muscle

**Figure 1:**
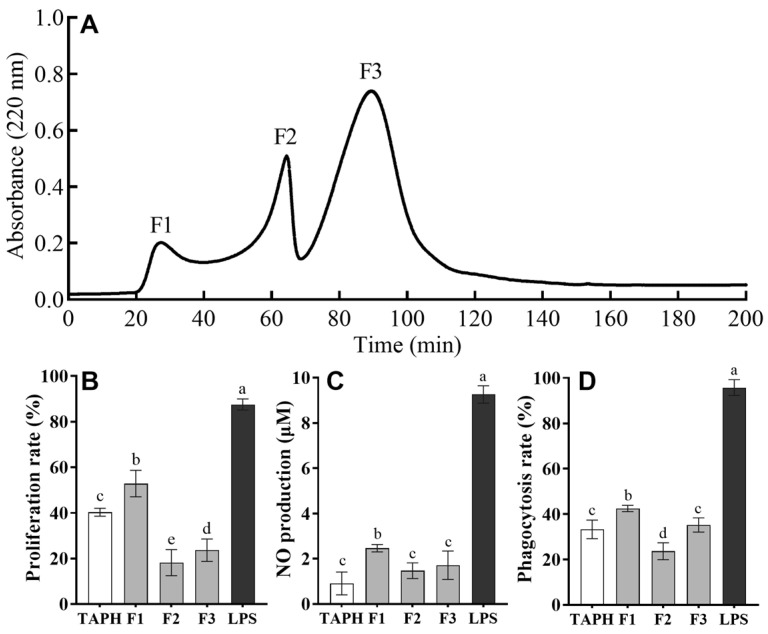
Elution curve of the peptide fractions obtained after the separation of trypsin hydrolysates of the *T. albacares* dark muscles using gel filtration and their effects on the immunomodulatory activity of RAW 264.7 cells. (**A**) Chromatogram profiles of TAPHs by Sephadex G-25 gel filtration and detection at 220 nm. The effects of gel filtration fractions on proliferation rate (**B**), NO production (**C**), and phagocytosis rate (**D**) in RAW 264.7 cells. The data are presented as means ± SD. Different lower-case letters indicate significant differences (*p* < 0.05) among the groups.

In this study, TAPHs were initially fractionated by size exclusion chromatography using a Sephadex G-25 column. As shown in Figure 1A, there were three major absorbance peaks at 220 nm (marked as F1, F2, and F3) according to their molecular weights (Figure 1A). These fractions were collected and lyophilized. To identify the presence of immunomodulatory peptides in TAPHs and three fractions after gel chromatography, different assays, such as cell proliferation, NO production, and phagocytosis, were used by using macrophage RAW 264.7 cells (Figure 1B–D). The evaluation of these parameters revealed significant differences among the three fractions, with fraction F1 demonstrating the highest immunomodulatory activity. Specifically, fraction F1 consistently exhibited significantly higher activity than F2, F3, and crude TAPH (*p* < 0.05) but lower activity than LPS in proliferation, NO production, and phagocytosis rates. Macrophages are pivotal in both innate and adaptive immunity, serving as essential defenders against pathogens. Their proliferation indicates immune system activation in response to threats [25], while their production of NO via the inducible nitric oxide synthase pathway effectively neutralizes microbial invaders [26]. Additionally, macrophages perform phagocytosis, a process enhanced by NO and other reactive species, to eliminate pathogens [27,28]. These findings indicated that the peptides with the lowest molecular weights had the highest immunomodulatory activity in the F1 fraction, which was consequently chosen for further purification.

**Figure 2:**
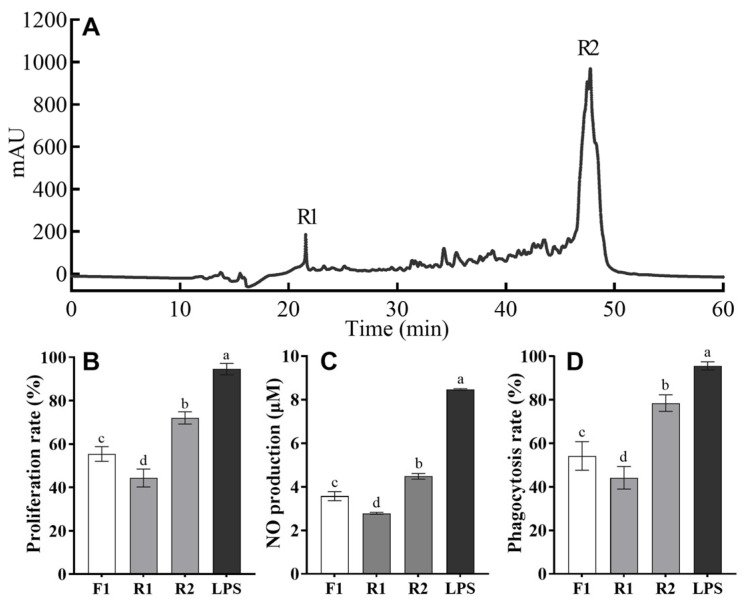
Elution curve of the fraction F1 separated in RP-HPLC and the effects of the R1 and R2 fractions on the immunomodulatory activity of RAW 264.7 cells. (**A**) Chromatogram profiles of TAPH by RP-HPLC and detection at 220 nm. The effects of HPLC fractions on proliferation rate (**B**), NO production (**C**), and phagocytosis rate (**D**) of RAW 264.7 cells. The data are presented as means ± SD. Different lower-case letters indicate significant differences (*p* < 0.05) among the groups.

RP-HPLC is an excellent technique for the separation of bioactive peptides from mixtures, given its high sensitivity, excellent resolution, and reliable reproducibility [26]. In the present study, fraction F1 derived from gel filtration, which had the highest immunomodulatory peptide activity, was further fractionated using RP-HPLC on a semi-preparative C18 column. The elution profile of F1 is illustrated in Figure 2A. Two main fractions, R1 and R2, were separately collected and subsequently lyophilized. The effects of F1, R1, and R2 on cell proliferation rate, NO production, and phagocytosis rate were also examined. As shown in Figure 2B–D, both R1 and R2 exhibited a certain degree of immunomodulatory activities. Notably, fraction R2 significantly outperformed fraction R1 and F1 (*p* < 0.05), stimulating the proliferation of RAW 264.7 cells to about 72.04%, the phagocytosis rate to 78.5%, and the NO production to 4.48 μM. In contrast, fraction R1 showed a lower proliferation rate than fraction F1, with no significant difference in phagocytosis rate and NO production.

It has been reported that naturally occurring immunomodulatory peptides are typically hydrophobic [27], which correlates with a longer retention time in RP-HPLC. In this study, fraction R2, which exhibited superior immunomodulatory activity, also had a much longer retention time (47.76 min) than R1 (21.57 min). This finding is consistent with a previous study that purified immunomodulatory peptides from *Cyclina sinensis* hydrolysates [28]. Therefore, fraction R2 was expected to contain a higher concentration of immunomodulatory peptides and was selected for further analysis of amino acid sequences.

### 3.3. Identification of Peptides

The amino acid sequences of the R2 fraction were identified by LC-MS/MS. The peptides identified through de novo sequencing by Peak Studio 10.6 software are presented in Table 1. A total of 17 peptides with ALC scores greater than 90 were identified from the fraction. These sequences were further examined in the BIOPEP-UWM database [29], and no matches were found, indicating that they are all novel peptides.

Their length, molecular weights, hydrophobicity, and isoelectric point (pI) were calculated using an online tool (http://www.pep-calc.com/, accessed on 20 August 2024, [30]). Briefly, the length of the identified peptide ranged from 5 to 12 amino acids, with molecular weights varying from 616 to 1441 Da. Although food-derived peptides with immunomodulatory effects are reported to be short (2–10 residues), their lengths were also closely related to their sources [27]. For instance, some immunomodulatory peptides identified from aquatic foods, such as *Crassostrea hongkongensis* [31] and *Cyclina sinensis* [28], also contain more than 10 residues, which aligned with the results of this study.

Furthermore, peptides with a higher percentage of hydrophobic amino acids in their sequences are considered to possess better immunomodulatory activity [27]. This is because hydrophobic amino acids contribute to the preliminary interaction between the peptides and cell membrane, improving immune regulation [28,32]. In this study, six peptides (No. 2, 12–16) were found to contain multiple hydrophobic amino acids, with a ratio of 50% or more, suggesting their potential for immunomodulatory effects. However, exceptions have been also reported. For example, Yu et al. [33] isolated and purified an immunologically active peptide from *Hericium erinaceus* with the amino acid sequence KSPLY. Despite containing only one hydrophobic amino acid, the peptide exhibited substantial immunomodulatory activity. Hence, further investigation was needed to confirm their possibilities of being immunomodulatory active. In this study, a computation-assisted approach was employed to predict and screen for the most potent ones.

### 3.4. Screening of Peptides via Molecular Docking

Molecular docking-based techniques have revolutionized the screening process for potential compounds from a vast pool of molecules by computing their affinity towards a receptor of interest [34]. In this study, we selected TLR2 and TLR4/MD-2 as the target receptors based on previous studies demonstrating their involvement in immunomodulatory peptide recognition on RAW 264.7 cells [35,36]. The 17 peptides identified above were docked into the binding pockets of TLR2 and TLR4/MD-2. All peptides were successfully docked with TLR2 and TLR4/MD-2, and the docking results are presented in Table 1. More negative values represent lower binding energy of ligands–receptor interaction, indicating a stronger binding affinity. Generally, the 17 peptides with varying physicochemical properties (molecular masses of 616.32–1441.69 Da, hydrophobicity of 27.27–55.56%, and pI values ranging from 3.84 to 6.17) that were tested exhibited slightly stronger binding affinities with TLR2 (binding scores from −7.71 to −12.21 kcal/mol) compared to TLR4/MD-2 (binding scores from −7.69 to −11.19 kcal/mol). This pattern was observed in most peptides, with notable exceptions being peptides No. 3, 7, 14, 16, and 17, where TLR4/MD-2 binding was stronger. Notably, the peptide YGSVELDELGK (No. 8) showed the strongest bind affinity with both TLR2 (−12.21 kcal/mol) and TLR4/MD-2 (−10.22 kcal/mol), followed by peptides YGPNDNFFEGK (No. 17), HDCDLLR (No. 1), and FPPDYLDDALR (No. 14).

The significant binding affinities observed between our peptides and TLR2/TLR4-MD-2 complexes align with the known immunological roles of these receptors. TLR2 could recognize a diverse set of pathogen-associated molecular patterns, including lipopeptides from bacteria, fungi, parasites, and viruses, while TLR4, in complex with MD-2, is best known for its ability to recognize lipopolysaccharide (LPS), a major component of the outer membrane of Gram-negative bacteria. Following pathogen-associated molecular pattern recognition, TLR2 and TLR4 activate intracellular signaling pathways that ultimately lead to the production of inflammatory mediators and the initiation of an immune response [37,38]. Our molecular docking results suggest that the selected peptides, particularly YGSVELDELGK (No. 8), may interact with these receptors in a manner that could modulate these signaling pathways. The differential binding affinities observed between TLR2 and TLR4/MD-2 for certain peptides like FPPDYLDALR (No. 14) may indicate potential receptor selectivity, which could be advantageous for targeting specific immune pathways. To maximize their potential efficacy in interacting with both receptors, six peptides (No. 8, 17, 1, 14, 12, 4) were chosen based on the highest combined binding affinities to both TLR2 and TLR4/MD-2. These selected peptides were then synthesized to confirm their immunomodulatory activities in vitro.

### 3.5. Immunomodulatory Activity of Synthetic Peptides

The validation of the immunomodulatory activities of the selected peptides with the highest affinities to TLR2 and TLR4/MD-2 was performed through the evaluations of IL-6 and TNF-α production, along with proliferation rate of RAW 274.7 cells, NO production, and phagocytosis ability after exposing the cells to the synthetic peptides. The results are presented in Table 2. In general, all the peptides effectively increased the proliferation rate, phagocytosis ability, and NO, IL-6, and TNF-α production. Particularly, peptides HDCDLLR (No. 1) and YGSVELDELGK (No. 8) demonstrated significantly superior effects compared to the other peptides, with peptide HDCDLLR having a significantly higher proliferation rate, phagocytosis ability, and NO production than YGSVELDELGK (*p* < 0.05), whereas YGSVELDELGK significantly promoted more IL-6 and TNF-α secretion than HDCDLLR (*p* < 0.05) without compromising the normal functions of RAW 264.7 cells. These results were also consistent with their predicted binding affinity to the two receptors, where the binding score of YGSVELDELGK was higher than HDCDLLR (Table 1). The rest of the peptides demonstrated similar activities in terms of IL-6 and TNF-α production. However, there were notable exceptions. The peptide FPPDYLDDALR (No. 14) showed superior activity in terms of NO production. Meanwhile, the peptide QYGDLSTPEALK (No. 4) outperformed the others in terms of its ability to stimulate phagocytosis.

Cytokines such as IL-6 and TNF-α have low molecular weight and are soluble proteins synthesized and secreted by activated immune cells, such as macrophages, T lymphocytes, B lymphocytes, and NK cells [39]. Both IL-6 and TNF-α are involved in the pathogenesis of cardiometabolic syndrome, a cluster of conditions including obesity, insulin resistance, and cardiovascular disease, which makes them valuable markers of immunomodulatory activity [40]. The significant induction of these cytokines, particularly by YGSVELDELGK (No. 8), suggests that this peptide may have potential applications in modulating immune responses related to these conditions. Overall, all these findings indicated that the peptides HDCDLLR and YGSVELDELGK had potent stimulatory effects, while the rest of the peptides were less effective at activating these cellular functions.

### 3.6. In Silico Molecular Docking of HDCDLLR and YGSVELDELGK

**Figure 3:**
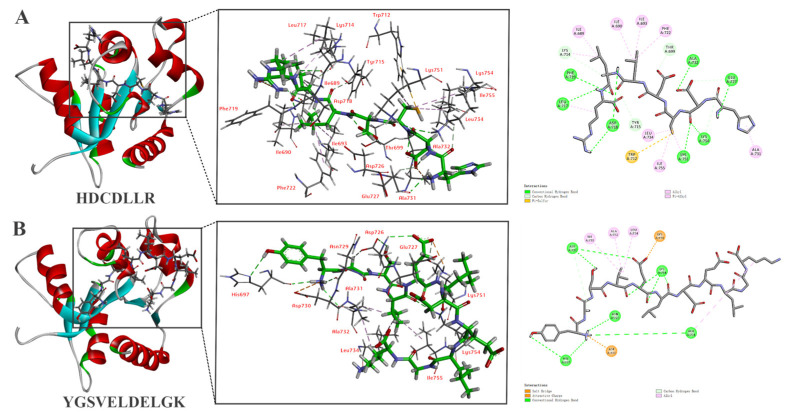
The molecular interaction of (**A**) HDCDLLR and (**B**) YGSVELDELGK binding to TLR2. The 3D reconstruction offers a panoramic view of the post-docking complex (**left**) and details the specific residues interacting with the peptide (**middle**), and the 2D representation highlights the site of peptide interaction with the TLR2 receptor (**right**).

**Figure 4:**
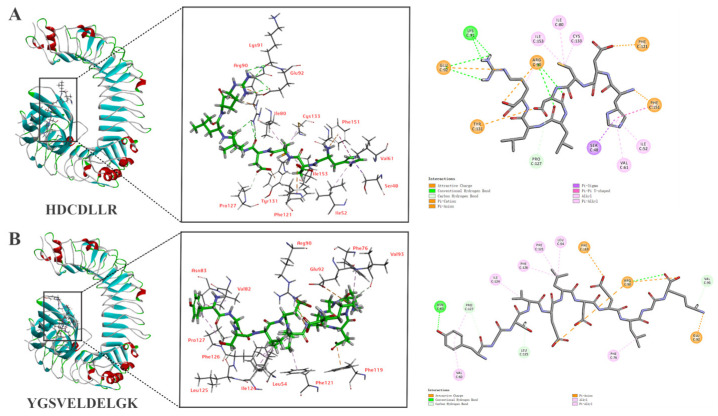
The molecular interaction of (**A**) HDCDLLR and (**B**) YGSVELDELGK binding to TLR4/MD2. The 3D reconstruction offers a panoramic view of the post-docking complex (**left**) and details the specific residues interacting with the peptide (**middle**), and the 2D representation highlights the site of peptide interaction with the TLR4/MD2 receptor (**right**).

The molecular mechanism of immunomodulatory activity was explored by analyzing the interaction between two peptides and the active sites of TLR2 and TLR4/MD-2. The docking stimulations of HDCDLLR and YGSVELDELGK with TLR2 and TLR4/MD-2 are shown in Figure 3 and Figure 4. Both peptides demonstrated strong bonding to the active sites of two receptors through different types of interactions, suggesting that the immunomodulatory activities of peptides are predicated on their interaction with the two receptors.

The docking result of HDCDLLR with TLR2 revealed a total of 24 interactions, including 13 hydrogen bonds, 10 hydrophobic interactions, and 1 π/sulfur interaction (Figure 3A). Hydrogen bonds were observed at the residues Glu727, Asp718, Leu717, Asp718, Phe719, Ala732, Lys751, Lys754, Thr699, Tyr715, Glu727, and Lys754 with bond distances typically ranging from 1.98 to 2.67 Å. Ten hydrophobic interactions were found between HDCDLLR and Leu734, Lys751, Ile755, Ile690, Ile693, Ile689, Ile717, Lys714, Ala731, and Phe722 residues of TLR2. A unique π/sulfur interaction was also observed between the peptide and Trp712 of TLR2. Meanwhile, YGSVELDELGK formed 11 hydrogen bonds with residues Asn729, Asp726, Ala731, Lys754, His697, Asp726, Lys751, Lys751, and Asp726; two electrostatic interactions with residues Lys751 and Asp730; and four hydrophobic interactions with residues Ala731, Ala732, Leu734, and Ile755 (Figure 3B).

The docking of HDCDLLR and TLR4/MD-2 complex revealed nine hydrogen bonds (with residues Arg90, Glu92, Lys91, and Pro127), five electrostatic interactions (with residues Arg90, Glu92, Phe151, Phe121, and Tyr131), and eight hydrophobic interactions (with residues Ser48, Phe151, Arg90, Cys133, Ile80, Ile153, Ile52, and Val61) (Figure 4A). Concurrently, YGSVELDELGK established five hydrogen bonds (with residues Asn83, Arg90, Pro127, Leu125, Val93, and Phe119), four electrostatic interactions (with residues Arg90 and Lys11), and seven hydrophobic interactions (with residues Leu54, Ile124, Phe76, Phe121, Phe126, Val82, and Pro127) on the C domain of TLR4/MD-2 (Figure 4B).

Hydrogen bond interactions are vital determinants for binding affinity and play a pivotal role in the stabilization of the ligand–protein complex. Our docking results predominantly revealed hydrogen bonds, which could potentially enhance the stimulation of immune activity. Notably, the higher number of hydrogen bonds and overall molecular interactions observed between HDCDLLR and TLR2 (24 total interactions, including 13 hydrogen bonds) compared to YGSVELDELGK (17 total interactions, including 11 hydrogen bonds) can be attributed to HDCDLLR’s unique structural characteristics. The presence of charged residues (His, Asp) and the cysteine residue in HDCDLLR may facilitate more extensive hydrogen bonding network interaction with Trp712 of TLR2 [41]. Additionally, the compact size of shorter peptides may allow for deeper penetration into TLR2 binding pockets, resulting in more stable complexes through multiple interaction types. Overall, the binding of two peptides, HDCDLLR and YGSVELDELGK, to the active sites of TLR2 and TLR4/MD-2 through hydrogen bonds and non-bond interactions may trigger the cellular signal transduction pathway, thereby activating the body’s immune response.

## 4. Conclusions

In this study, a series of immunomodulatory peptides were purified from TAPHs using gel filtration and RP-HPLC, and their amino acid sequences were identified using mass spectrometry. In silico analysis was performed to screen out the peptides with better immunomodulatory activities. A total of six peptides were selected, synthesized, and had their immunomodulatory activities verified by measuring their proliferation rate, phagocytosis rate, and NO, IL-6, and TNF-α production. Notably, two peptides, HDCDLLR and YGSVELDELGK, significantly promoted the proliferation and phagocytosis rate of RAW 274.4 cells, also inducing the secretion of NO, IL-6, and TNF-α. Further molecular docking analysis revealed that the binding of HDCDLLR and YGSVELDELGK to the active sites of TLR2 and TLR4/MD-2 through hydrogen bonds and non-bond interactions may stimulate macrophage activation. These findings suggested that trypsin hydrolysates of yellowfin tuna meat possess potential immunomodulatory activity. The peptides HDCDLLR and YGSVELDELGK could serve as potential immune enhancers derived from natural food sources.

## Figures and Tables

**Table 1:** Physicochemical properties and receptor binding affinities of bioactive peptides identified in the R2 fraction isolated by RP-HPLC.

No.	Sequence	Score	Molecular Mass (Da)	Hydrophobicity (%)	pI	Bind Score (kcal/mol)	
						TLR2	TLR4/MD-2
1	HDCDLLR	99	870.98	28.57	5.21	−9.47	−9.45
2	FVNDEAFLR	99	1109.55	55.56	4.19	−8.02	−7.95
3	LGEDFLR	99	848.44	42.86	4.19	−7.71	−7.98
4	QYGDLSTPEALK	98	1320.66	33.33	4.19	−9.21	−8.87
5	CDLDLR	98	733.34	33.33	4.12	−7.93	−7.69
6	LEAER	97	616.32	40.00	4.25	−9.33	−8.19
7	LGGGEFLELR	97	1089.58	40.00	4.25	−7.76	−9.04
8	YGSVELDELGK	96	1208.59	27.27	3.92	−12.21	−10.22
9	EDPHLVPCLGTK	96	1307.65	41.67	5.23	−8.28	−9.4
10	LVYPNDNFFEGK	95	1441.69	41.67	4.19	−8.86	−8.7
11	ACGAEEFLKR	94	1122.55	40.00	6.17	−9.61	−8.01
12	APCEFNLK	94	920.44	50.00	6.05	−9.27	−9.16
13	DRLDTPLPDRPF	94	1440.74	50.00	4.32	−8.86	−8.56
14	FPPDYLDDALR	94	1320.64	54.55	3.84	−8.30	−10.22
15	QDPEDVLLSAFK	94	1360.69	50.00	3.88	−8.17	−8.54
16	AFDDAFAEFQR	93	1315.58	54.55	3.88	−7.91	−9.10
17	YGPNDNFFEGK	91	1286.56	27.27	4.19	−10.10	−11.19

**Table 2:** Immunomodulatory effects of synthesized peptides on proliferation, NO production, phagocytosis, and cytokine secretion of RAW 274.7 cells.

Treatment	Peptide Sequences	Proliferation Rate (%)	NO (μM)	Phagocytosis Rate (%)	IL-6 (pg/mL)	TNF-α (pg/mL)
Control	N/A	N/A	N/A	N/A	21.64 ± 2.05 ^e^	129.02 ± 11.89 ^f^
No. 1	HDCDLLR	68.81 ± 8.48 ^b^	7.02 ± 0.20 ^b^	88.61 ± 3.52 ^a^	74.27 ± 1.09 ^c^	511.86 ± 15.49 ^c^
No. 4	QYGDLSTPEALK	51.04 ± 3.60 ^d^	2.51 ± 0.32 ^e^	71.67 ± 2.74 ^c^	49.88 ± 2.19 ^d^	363.82 ± 8.47 ^e^
No. 8	YGSVELDELGK	51.58 ± 3.96 ^cd^	5.93 ± 0.30 ^c^	84.21 ± 3.76 ^b^	93.26 ± 0.62 ^b^	643.95 ± 9.05 ^b^
No. 12	APCEFNLK	54.00 ± 4.62 ^c^	2.57 ± 0.53 ^e^	28.83 ± 5.05 ^f^	49.03 ± 0.40 ^d^	384.85 ± 45.96 ^e^
No. 14	FPPDYLDDALR	45.51 ± 2.42 ^e^	5.00 ± 0.35 ^d^	49.94 ± 6.23 ^d^	54.11 ± 0.32 ^d^	401.13 ± 30.50 ^de^
No. 17	YGPNDNFFEGK	45.15 ± 6.83 ^de^	2.60 ± 0.70 ^e^	39.92 ± 7.64 ^e^	50.41 ± 0.64 ^d^	435.38 ± 4.66 ^d^
LPS	N/A	95.90 ± 0.37 ^a^	9.50 ± 0.41 ^a^	92.08 ± 1.82 ^a^	158.36 ± 7.34 ^a^	916.40 ± 24.16 ^a^

Values are expressed as mean ± SD; superscript letters a, b, c, d, e, and f are significantly different across columns (*p* < 0.05).

## Data Availability

The original contributions presented in this study are included in the article; further inquiries can be directed to the corresponding authors.
